# Oxidative Stress Measurement in Frozen/Thawed Human Sperm: The Protective Role of an In Vitro Treatment with Myo-Inositol

**DOI:** 10.3390/antiox11010010

**Published:** 2021-12-22

**Authors:** Rosetta Ponchia, Annunziata Bruno, Asia Renzi, Claudia Landi, Enxhi Shaba, Francesca Paola Luongo, Alesandro Haxhiu, Paolo Giovanni Artini, Alice Luddi, Laura Governini, Paola Piomboni

**Affiliations:** 1 Department of Molecular and Developmental Medicine, University of Siena, 53100 Siena, Italy; ponchia2@student.unisi.it (R.P.); bruno20@student.unisi.it (A.B.); asia.renzi@student.unisi.it (A.R.); francescapaola.lu@student.unisi.it (F.P.L.); alesandro.haxhiu@student.unisi.it (A.H.); paola.piomboni@unisi.it (P.P.); 2 Department of Life Sciences, University of Siena, 53100 Siena, Italy; landi35@unisi.it (C.L.); Enxhi.shaba@unisi.it (E.S.); 3 Department of Experimental and Clinical Medicine, Division of Obstetrics and Gynecology, Pisa University, 56100 Pisa, Italy; pgartini@gmail.com

**Keywords:** human sperm, myo-inositol, sperm motility, sperm cryopreservation, mitochondrial respiration, carbonyl groups

## Abstract

Despite its widespread use, sperm cryopreservation induces serious detrimental alterations in sperm function; indeed, it is commonly associated with decreased sperm viability and motility, and DNA fragmentation. Mechanisms of human sperm cryodamage are thought to be multifactorial, but oxidative stress seems to have a prominent role. A huge amount of data supported the cryoprotective effect of different antioxidants able to minimize the detrimental effects of reactive oxygen species (ROS) and improve the quality of spermatozoa. Among others, myo-inositol is one of the most powerful and has been reported to be effective in improving sperm quality and motility when used both in vivo and in vitro. This study aimed to determine the in vitro impact of myo-inositol in ameliorating sperm oxidative status during sperm cryopreservation. In particular, we demonstrated a significant improvement of sperm parameters (vitality and motility) when myo-inositol was added after sperm thawing (*p* < 0.05). Moreover, we showed that myo-inositol induces a significant increase in oxygen consumption, the main index of oxidative phosphorylation efficiency and ATP production. Finally, by means of 2D-electrophoresis, we demonstrated a significant decrease in the level of carbonyl groups, the main structural changes occurring in conditions of oxidative stress (*p* < 0.05). In conclusion, the sperm cryopreservation procedure we developed, assuring the reduction of ROS-induced sperm modifications, may improve the in vitro procedure currently used in ART laboratory for sperm cryostorage.

## 1. Introduction

Sperm cryopreservation is an effective strategy to preserve male fertility for those men undergoing chemotherapy, radiotherapy, and testicular surgery, as well as a procedure commonly adopted in Assisted Reproductive Technique (ART) laboratories to provide a viable therapeutic option in the field of assisted reproduction [1,2]. Unfortunately, cryopreservation requires freezing and thawing steps that are detrimental to the sperm membrane, acrosome, and DNA integrity, as well as sperm motility and vitality [3,4]. This is confirmed by the fact that only 50% of spermatozoa survive the freezing and thawing process [5]. Cryopreservation induces cryo- and osmotic stress, which in turn increases the rate of production of reactive oxygen species (ROS), as well as an alteration in antioxidant defence systems [6]; when the levels of ROS exceed the cell’s physiological antioxidant defences, a condition of oxidative stress (OS) is established.

Some distinctive sperm features, including the high polyunsaturated fatty acid (PUFA) content in the membranes, the large number of mitochondria, and the reduced antioxidant content in the cytoplasm, make male gametes more susceptible to ROS damage [7]. Of some note, the abnormal concentrations of ROS may be detected in 30–80% of infertile men, and that seminal ROS are negatively correlated with several sperm fertility parameters suggests that oxidative stress is a key factor in male infertility [8]. Despite this, low levels of ROS have an important physiologic role since they are fundamental for sperm maturation, hyperactivation, capacitation, and acrosome reaction [6]. The main endogenous source of ROS is mitochondrial oxidative phosphorylation (OXPHOS)—the process assuring the large part of cellular ATP. Sperm motility is ATP-dependent, so glycolysis and, to a greater extent, mitochondrial respiration are critical for sperm function. During the production of ATP by oxidative phosphorylation, mitochondria oxidize different substrates, thereby reducing oxygen to water. Therefore, the oxygen consumption over time, measured by an oxygraphic assay, is an indirect but worthwhile indicator of mitochondrial functionality [9]. Mitochondrial functionality of sperm can be largely affected by ROS and therefore, oxygen consumption is a good biomarker of intracellular OS.

In order to decrease ROS production, many studies suggested the beneficial effect of administering some antioxidant compounds, which are the main defence factors against OS induced by ROS [5,10,11,12,13]. Recently, our research group demonstrated that an in vitro treatment with myo-inositol is effective in protecting sperm against oxidative damage to DNA [14]. Moreover, we demonstrated that myo-inositol is also efficient in increasing sperm motility and oxygen consumption, which is the main index of oxidative phosphorylation efficiency and ATP production [14]. Another attractive strategy to indirectly measure OS is to analyse the oxidative damage to proteins. Indeed, carbonyl groups are produced when protein side chains are oxidized. In oxidative stress conditions, carbonylated proteins are early and relatively stable markers, which are very effective in the measurement of OS [15]. This is especially true for sperm proteins, since male gametes are transcriptionally and translationally inactive; this fact reduces the complexity of the sperm proteome analysis [16].

On these bases, the objectives of this study were: (1) to determine the effect of an in vitro myo-inositol treatment before or after freezing and thawing procedures on the main semen parameters (sperm vitality and motility), as well as on the mitochondrial oxygen consumption, and (2) to measure, by means of a proteomic approach, the direct effects of OS on proteins through the analysis of carbonyl groups as specific and sensitive markers of oxidative stress.

## 2. Materials and Methods

### 2.1. Samples Collection

The study was conducted on 25 normozoospermic semen samples, selected according to the WHO (2010) criteria [17]. The ejaculate samples were collected, by masturbation, in sterile containers after a period of abstinence between 2 and 5 days. The study was approved by the Ethics Committee of the Siena University Hospital (approval ID: CEAVSE 191113).

### 2.2. Samples Treatment

Seminal analysis was carried out, within 30 min after fluidification, by two independent blinded observers (obtained data are the mean value of the two observations). The volume, viscosity, pH, and appearance of the semen were evaluated together with sperm concentration, progressive and non-progressive motility, and morphology. Sperm concentration was evaluated using a Makler chamber (CooperSurgical Fertility Solutions, Målov, Denmark) with 10 µL of appropriately diluted sample and observed through an optical microscope at 200× magnification. A Makler counting chamber measures 1 × 1 mm and is divided into 100 squares, each of which is 0.1 × 0.1 mm. The concentration was established by counting sperm in 10 squares and the obtained number expresses the concentration in 10^6^/mL. Sperm morphology was evaluated in the optical microscope using pre-coloured glasses.

Sperm vitality was evaluated through an eosin Y test. Samples of 10 µL were mixed with 10 µL of eosin Y solution, and the sperm were observed in the optical microscope at 400× magnification, which followed WHO 2010 parameters [17]. Dead sperm appeared pink-coloured, while alive sperm appeared uncoloured. The vitality test was performed in all samples before and after myo-inositol treatment to exclude any negative impact on spermatozoa.

**Scheme 1:**
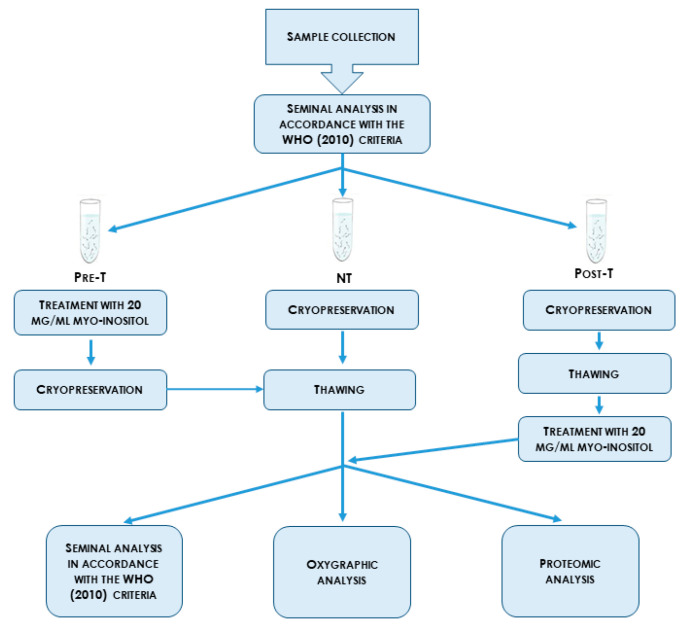
Design of the study. Pre-T: pre-cryopreservation myo-inositol treated samples; NT: cryopreserved samples without myo-inositol; Post-T: post-cryopreservation myo-inositol treated samples.

In order to evaluate the effect of myo-inositol treatment on cryopreservation, 25 sperm samples were divided into three aliquots: cryopreserved samples without myoinositol (NT; *n* = 25), pre-cryopreservation myo-inositol treated samples (Pre-NT; *n* = 25), and post-cryopreservation myo-inositol treated samples (Post-T; *n* = 25). Each aliquot, containing approximately 45 million sperm, was centrifuged at 800× *g* for 10 min. The in vitro treatment with myo-inositol was carried out for 20 min at room temperature with the final concentration of myo-inositol being 20 mg/mL. The NT, Pre-T, and Post-T samples were cryopreserved with cryoprotectant 1:1 *v*:*v* (Freezing Medium, Fujifilm, Rome, Italy) by using a manual method. Briefly, straws containing sperm were firstly placed at –20 °C for 30 min and then moved into a goblet in a mixture of liquid nitrogen vapour before being placed in liquid nitrogen. Sperm samples were thawed by placing them immediately at 37 °C. After complete thawing, the cryoprotectant was removed by adding the culture medium before centrifugation for 10 min at 500× *g*. The flowchart of this study is summarized in Scheme 1.

### 2.3. Hypotonic Swelling and Oxygraphic Assay

All the NT, Pre-T, and Post-T sperm samples were demembranated by hypotonic swelling treatment. First, samples were washed two times by resuspension in isotonic salt medium with pH 7.4 (113 mmol/L KCl, 12.5 mmol/L of KH_2_PO_4_, 2.5 mmol/L of K_2_HPO_4_, 3 mmol/L of MgCl_2_, 0.4 mmol/L of ethylene diamine tetra-acetic acid (EDTA), and 20 mmol/L of Tris adjusted to pH 7.4 with HCl) and centrifuged for 10 min at 800× *g*. Spermatozoa were then subjected to hypotonic treatment by adding in ice-chilled hypotonic medium (potassium phosphate 10 mml/L, pH 7.4, with 2 g/L BSA, bovine serum albumin) for 90 min, as reported in the literature [14,18]. Sperm were then washed three times using the isotonic salt medium at pH 7.4 to proceed with an oxygraphic assay [14,18]. A Clark-type oxygen probe (Hansatech Oxygraph, Norfolk, UK) was used to measure the oxygen uptake by spermatozoa at 36 °C. To this end, demembranated sperm cells were vigorously stirred in the reaction chamber in an isotonic salt medium without EDTA and were temperature equilibrated. The rate of oxygen uptake by sperm was expressed as nmol O_2_·mL^−1^·min^−1^. The oxygen consumed by the sperm was determined as the difference between the oxygen present at the time of the addition of the spermatozoa and that present after 10 min. To confirm that O_2_ consumption was due to mitochondrial OXPHOS, selective inhibitors of mitochondrial OXPHOS complexes were used and their activity was evaluated through an oxygraphic assay, according to in-house validated protocols [18,19].

### 2.4. Samples Preparation for Two Dimensional Electrophoresis (2DE) and Redox Proteomic Analysis

The pellets of NT, Pre-T, and Post-T sperm samples were diluted in 7 M Urea, 2 M Thiourea, 4% (*w*/*v*) 3-[(3-cholamidopropyl)dimethylammonio]-1-propanesulfonate (CHAPS), 1% (*w*/*v*) dithioerythritol (DTE), 0.5% (*p*/*v*) Triton X-100 and left in ice for 2 h. Once thoroughly vortexed, samples were briefly centrifuged at 15,000× *g* and total protein quantization was determined according to the Bradford method [20]. Sixty micrograms of proteins for the samples were loaded onto immobilized pH gradient (IPG) strips for the analytical run. One hundred and twenty micrograms of proteins were cup-loaded onto IPG strips for the redox proteomic analysis.

### 2.5. Two-Dimensional Electrophoresis

Isoelectric focusing was performed using the immobiline–polyacrylamide system, as described [21,22] with a non-linear wide-range immobilized pH gradient (pH 3–10; 18 cm long IPG strips; Cytiva, Uppsala, Sweden) using an Ettan™ IPGphor™ system (GE Healthcare, Chicago, IL, USA). IPG strips were rehydrated with 350 μL of sample and 0.2% (*v*/*v*) carrier ampholyte for the analytical and transfer runs at 16 °C with the following electrical conditions: 30 V for 8 h, 200 V for 2 h, from 200 V to 3500 V in 2 h, 3500 V for 2 h, from 3500 V to 5000 V in 2 h, 5000 V for 3 h, from 5000 V to 8000 V in 1 h, 8000 V for 3 h, from 8000 V to 10,000 V in 1 h, and 10,000 V for the rest of the run until it reached a total of 90,000 VhT. The second dimension was carried out on 9–16% polyacrylamide linear-gradient gels after the equilibration step [23]. As previously described, the analytical and transfer gels were stained with silver diaminohydroxide solution and then digitalized using an Image Scanner III laser densitometer and the corresponding LabScan 6.0 software (GE Healthcare, Chicago, IL, USA).

Melanie 9.0 software (Geneva Bioinformatics (GeneBio), Geneva, Swiss) was used to perform 2D imaging and data analysis of the relative percentage for spot volume.

All matched spots have been submitted to the XLStat software (version 2021.2, XLSTAT-life Science, Paris, France) to perform the Heatmap analysis, according to the Euclidean distance.

### 2.6. Redox Proteomics

After the isoelectric focusing, strips subjected to transfer for the western blot (WB) were derivatized to detect carbonyl groups by rinsing them with water and incubating them at room temperature in 5% trichloroacetic acid containing 2,4-dinitrophenylhydrazine (DNPH) at 10 mm for 20 min. The DNPH-treated strips were washed twice with a solution containing 8 M urea, 20% *v*/*v* glycerol, 1% *w*/*v* sodium dodecyl sulphate, and 150 mM Tris-HCl (pH 6.8) for 5 min [24,25] and then equilibrated as previously reported [23]. After equilibration and the second-dimension separation, proteins were transferred into a nitrocellulose membrane (Hybond ECL, GE Healthcare, Chicago, IL, USA) [26] stained with Ponceau Red (0.2% *w*/*v* Ponceau S in 3% *v*/*v* trichloroacetic acid) to check for correct protein transfer and equal protein loading. Membranes were incubated with rabbit polyclonal antibodies anti-2,4-dinitrophenol (DNP) (Sigma, St. Louis, MO, USA; dilution 1:10,000), washed, and then incubated with goat peroxidase-conjugated, anti-rabbit immunoglobulin G (Sigma, working dilution 1:7000). At the end, the ECL chemiluminescence detection system (GE Healthcare, Chicago, IL, USA) was used to detect the immunoreactive, chemiluminescent spots captured after different exposure times (3 min, 10 min, 20 min), and were analysed with ImageJ (1.51p, NIH, Chicago, IL, USA) to measure the spot intensity. The statistical significance was assessed.

### 2.7. Statistical Analysis 

A statistical analysis was performed by means of the GraphPad Prism 5.0 (GraphPad Software, San Diego, CA, USA) using nonparametric tests. The differences among groups of data were tested by the Kruskal–Wallis test or the Mann–Whitney test. The statistical significance was set at *p* < 0.05.

## 3. Results

### 3.1. Myo-Inositol In Vitro Treatment Increases Vitality and Progressive Motility of Cryopreserved Sperm

The main sperm parameters in fresh ejaculates are summarized in Supplementary Table S1, along with the parameters for cryopreserved sperm without myo-inositol treatment (NT), or with myo-inositol added before (Pre-T) or after (Post-T) cryopreservation.

**Figure 1:**
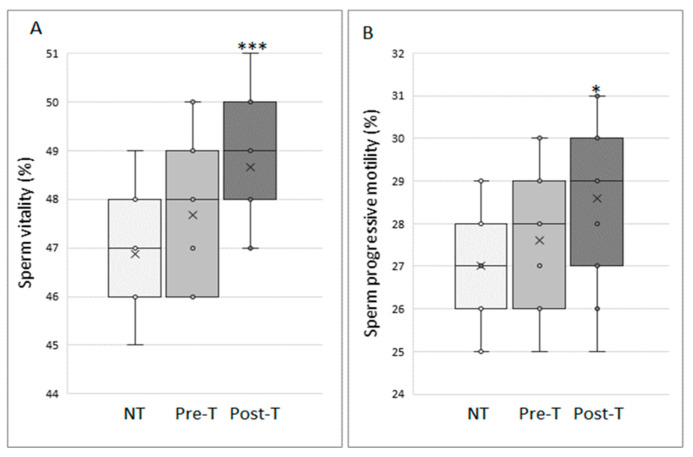
Sperm vitality (**A**) and sperm progressive motility (**B**) of cryopreserved sperm that are untreated (NT; *n* = 25), treated with 20 mg/mL myo-inositol for 20 min before (Pre-T; *n* = 25), or after (Post-T; *n* = 25) sperm cryopreservation. Graphical diagrams are plotted as box–whisker plots, where boxes show the interquartile range with median and mean values, and whiskers represent min and max confidence intervals. * *p* < 0.05; *** *p* < 0.001.

We demonstrate that myo-inositol treatment induces a significant improvement in sperm recovery and quality after the freezing –and thawing procedure (Scheme 1; Figure 1). Indeed, sperm vitality is increased in the treated sperm and this improvement is even more significant when the treatment is carried out after sperm cryopreservation (*p* < 0.001) (Figure 1A). Moreover, as shown in Figure 1B, a trend in progressive motility may be observed in Pre-T samples. Despite this, the statistical significance is not reached, while a significant increase of about 6% is present in samples processed after freezing/thawing (27% in untreated versus 29% treated samples; *p* < 0.01) (Figure 1B).

### 3.2. Myo-Inositol In Vitro Treatment Increases the Oxygen Consumption Rate of Cryopreserved Sperm

**Figure 2:**
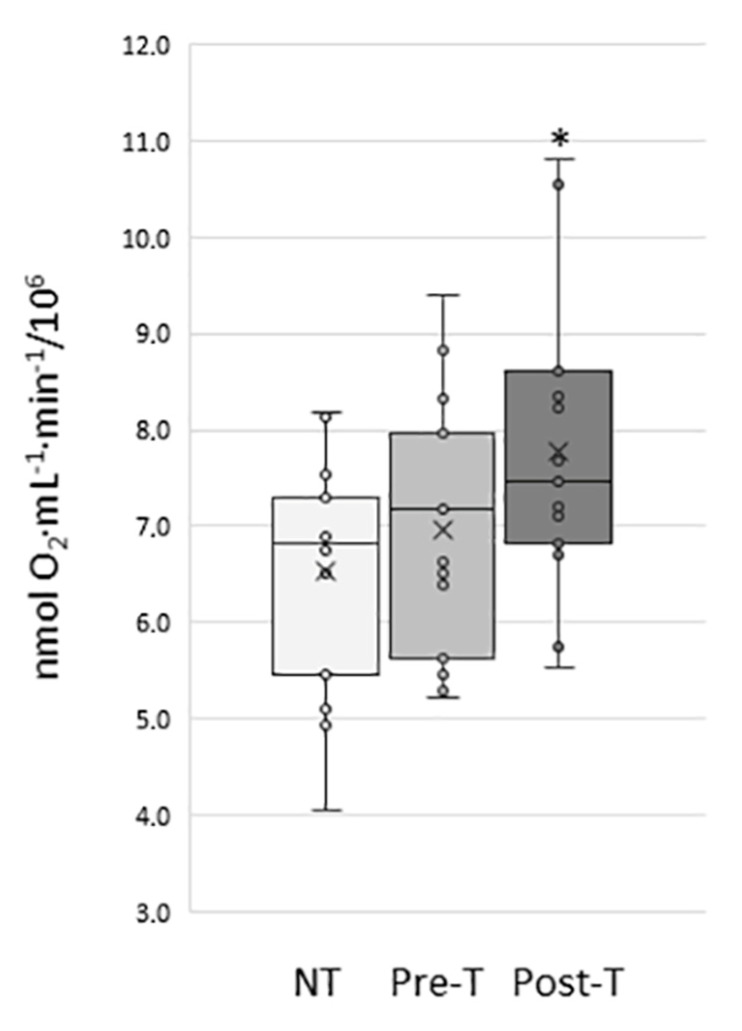
Oxygen consumption, expressed as nmol O_2_·mL^−1^·min^−1^, of cryopreserved sperm that are untreated (NT; *n* = 25), treated with 20 mg/mL myo-inositol for 20 min before (Pre-T; *n* = 25), or after (Post-T; *n* = 25) sperm cryopreservation. Graphical diagrams are plotted as box–whisker plots, where boxes show the interquartile range with median and mean values, and whiskers represent min and max confidence intervals. * *p* < 0.05.

The oxygen consumption rate was then assessed on the cryopreserved sperm samples treated before and after cryopreservation with 20 mg/mL of myo-inositol. We observed a significant increase in the oxygen consumption rate of treated sperm compared to NT (Figure 2). Moreover, a significant improvement in the oxygen consumption rate is shown between not-treated samples and post-treated samples, according to the progressive motility data.

### 3.3. Proteomic Analysis

**Figure 3:**
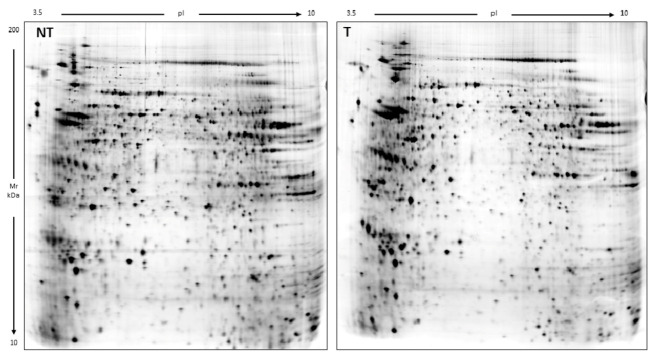
Master gels obtained by two-dimensional electrophoresis that are representative of the untreated and post-treated (NT and Post-T) sample groups. Two dimensional electrophoresis (2DE) maps show protein spots with an isoelectric point from pH 3 to pH 10 and a molecular weight from 10 to 200 kDa.

In order to investigate the significant differences that emerged in the total amounts of CO groups, proteins from the NT and Post-T samples were analysed by two-dimensional electrophoresis, followed by qualitative and semiquantitative analyses of the spots. A master gel was selected for each condition to match and compare them, and to look for quantitative protein differences. In this way, we demonstrated that the analysed dataset enumerated ~2114 protein spots *per* gel (Figure 3). The 2DE image comparison by Melanie 9 (version 9.2, Cytiva, Uppsala, Sweden) showed no evidence of any particular differences among the analyzed conditions.

**Figure 4:**
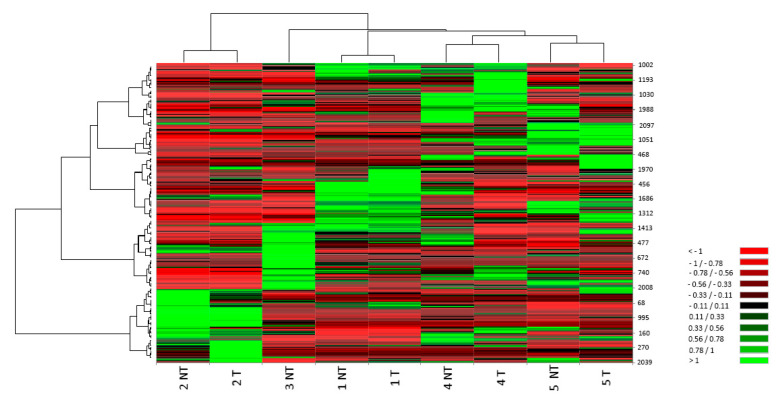
Heat maps: columns correspond to individual preparations of cryopreserved sperm that are untreated (NT) or treated with 20 mg/mL myo-inositol for 20 min after cryopreservation (Post-T), while rows correspond to matched spots. Colour scale (from high value in green to low value in red) illustrates % volume values of the spots.

The unsupervised analysis performed as a data quality control by the heatmap analysis shows that every not-treated (NT) sample clusters close to its treated counterpart (Post-T) (Figure 4), thereby highlighting that the treatment did not alter the protein composition of the spermatozoa samples.

**Figure 5:**
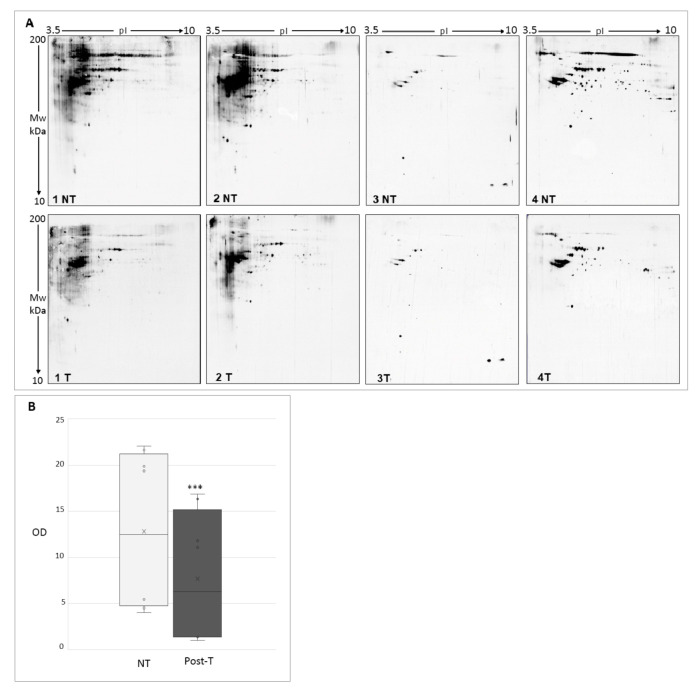
(**A**) Western blotting after two dimensional electrophoresis showing carbonyl groups in untreated (NT) and in the corresponding treated (Post-T) sample. Four sperm samples were analysed. (**B**) Computer-assisted semiquantitative analysis of the overall relative intensity of the carbonyl groups in NT and in the corresponding Post-T sample. Carbonyl groups intensity was measured (pixel/mm^2^). The graph highlights the significant decrease in carbonyl groups in Post-T samples. OD, optical density. Values are expressed as a mean (standard deviation). *** *p* < 0.01.

To test whether the improvements in seminal parameters and mitochondria respiratory activity induced by myo-inositol treatment are attributable to the antioxidant action of this compound, we quantified the level of carbonylated proteins as an indirect, but early and stable, biomarker of OS induced by the sperm cryopreservation procedure. We detected carbonylated proteins in the frozen and thawed sperm, which were coupled with two-dimensional high resolution electrophoresis with a western blot analysis applying methods for the detection of oxidized carbonylated proteins (derivatization with DNP and use of anti-DNP antibodies). Figure 5A shows that the redox proteomics analysis demonstrated a significant decrease in protein oxidation in sperm samples treated with 20 mg/mL myo-inositol for 20 min after cryopreservation. The main spot intensity was measured and graphed in Figure 5B; the graph quantifies the significant decrease in carbonyl groups in Post-T samples.

## 4. Discussion

Sperm cryopreservation and thawing are associated with increased ROS production, as well as decreased levels of antioxidant defences [27,28]. Therefore, the use of antioxidants is one of the most important challenges to overcome the cryodamage during sperm freezing and thawing. Indeed, antioxidant supplementation had been widely employed in different species to avoid cryodamage during sperm cryopreservation when the concentration of ROS exceeds the physiological limit [29].

Many recent studies have focused on evaluating the effect of myo-inositol on sperm motility and its potential antioxidant properties, both in vivo and in vitro [30,31]. First, we excluded the negative effects of the myo-inositol treatment on sperm cells with the vitality eosin Y test prior to any analysis, thus confirming the safety of this compound. Therefore, our experiments corroborate the positive influence of myo-inositol on sperm motility that previous literature data reported. Moreover, this work provides significant insight on the in vitro effects of myo-inositol in protecting sperm vitality and motility during cryopreservation. Our results confirm the positive influence that myo-inositol has on sperm motility, which are reported both in vivo and in vitro by other studies; indeed, a significant enhancement of the post-thaw sperm quality after in vitro myo-inositol supplementation of cryopreserved ejaculate sperm has been reported both in humans and in animals [32,33]. Interestingly, all these studies used myo-inositol supplementation before sperm cryopreservation, while our data demonstrated for the first time that myo-inositol supplementation is even more effective in protecting sperm vitality and progressive motility if added after sperm thawing rather than before sperm freezing.

The other important result from this study is the significant increase in the oxygen consumption rate in cryopreserved sperm supplemented with myo-inositol after thawing. Our results agree with the previous studies [14,18,34], demonstrating the role of OXPHOS in ATP production and the effect of myo-inositol on mitochondrial functionality [35,36]. Indeed, myo-inositol is able to act directly on the mitochondrial level, thereby increasing mitochondrial membrane potential (MMP), which is an important apoptotic marker frequently used as an indicator of male fertility and directly correlates to motility, sperm fertility potential, and embryo quality [37,38,39]. High levels of MMP can describe a good mitochondrial cristae integrity, and subsequent optimal levels of cellular activity and vitality. Moreover, as reported in the literature, mitochondrial OXPHOS has a predominant role in ATP production for sperm motility sustainment, with a definitively more important role than glycolysis [40]. Therefore, our study lets us hypothesize that the ability of myo-inositol to improve sperm mitochondrial function is related to its demonstrated capability to significantly improve sperm motility. Consequently, the oxygraphic analysis represents a powerful strategy to measure the mitochondrial activity and, indirectly, the sperm functionality.

We finally provided evidence for an indirect, but accurate, measurement of oxidative stress-induced damage on proteins [14,41]. It is known that ROS are able to oxidize the side chains of amino acids—most commonly the amino acids lysine, arginine, and proline—giving rise to reactive aldehydes and ketones known as protein carbonyls. These compounds are long lasting and stable, and that is why they are the most general indicator of oxidative protein damage [15,41,42]. 

By using this approach, we demonstrated a significant decrease in the level of carbonyl groups in cryopreserved sperm treated with myo-inositol after sperm thawing. This result is very important, since the analysis of protein modifications is an excellent strategy to study oxidative stress levels in biological samples as it overcomes the instability of ROS, which are continuously generated and dissolved [43]. These compounds are stable longer than ROS, which is why they are the most general indicator of oxidative damage to proteins. Moreover, several studies report that protein carbonylation should revert within 30 min, revealing that the process is transient [44,45]. Based on this knowledge and the need to reduce sperm handling time, we set up this protocol to protect sperm from freezing/thawing damage. 

Furthermore, we want to point out that we tested the effectiveness of this approach in normozoospermic patients and therefore, further studies are needed to extrapolate the results for oli-, astheno-, and/or teratozoospermic patients. Only then should the treatment with myo-inositol be used to improve the outcome of actual ARTs protocols, particularly in terms of idiopathic infertility. Therefore, such findings are interesting and may have important implications on the future outcomes of assisted reproductive techniques using cryopreserved sperm.

## Data Availability

Data is contained within the article or Supplementary Material.

## Supplementary Materials

The following are available online at https://www.mdpi.com/article/10.3390/antiox11010010/s1, Table S1: Main sperm parameters, in basal (fresh) or in cryopreserved sperm without myo-inositol treatment (NT), in sperm treated with myo-inositol before cryopreservation (Pre-T) and in sperm treated with myo-inositol after cryopreservation (Post-T).
