# Supplementary material for: Oxidative Stress Measurement in Frozen/Thawed Human Sperm: The Protective Role of an In Vitro Treatment with Myo-Inositol
Source: Antioxidants (Basel). 2021 Dec 22;11(1):10. doi: 10.3390/antiox11010010 (PMC8773045; doi:10.3390/antiox11010010)
Supplement: Supplementary file 1 [file antioxidants-11-00010-s001.zip › antioxidants-1480893-supplementary.pdf]

Table S1: Main sperm parameters, in basal (fresh) or in cryopreserved sperm without myo-inositol treatment (NT), in sperm treated with myo-inositol before cryopreservation (Pre-T) and in sperm treated with myo-inositol after cryopreservation (Post-T).

	**Fresh**		**NT**		**Pre-T**		**Post-T**	
	**mean**	**sd**	**mean**	**sd**	**mean**	**sd**	**mean**	**sd**
**Sperm number ($\times 10^6$/mL)**	196.0	27.5	43.5	4.6	43.7	4.8	44.2	4.6
**sperm motility**	63.7	3.0	27.0	1.5	27.6	1.6	28.5	1.8
**sperm vitality**	78.8	3.1	46.9	1.1	47.7	1.4	48.5	1.1
